# Recurrent Stroke-Like Symptoms After Cesarean Section Deliveries in a Female Patient With X-Linked Charcot-Marie-Tooth Type 1

**DOI:** 10.3389/fneur.2020.00008

**Published:** 2020-01-28

**Authors:** Qu Li, Chen Chen, Yan Ren, Xu Liu

**Affiliations:** ^1^Department of Neurology, First Affiliated Hospital of China Medical University, Shenyang, China; ^2^Key Laboratory of Cell Biology, Ministry of Public Health, Key Laboratory of Medical Cell Biology, Ministry of Education, The Research Center for Medical Genomics, School of Life Sciences, China Medical University, Shenyang, China

**Keywords:** X-linked Charcot-Marie-Tooth, *GJB1* gene, connexin 32, magnetic resonance imaging, central nervous system symptom

## Abstract

**Background:** X-linked Charcot-Marie-Tooth type 1 (CMTX1) is the second most frequent form of CMT, which is caused by mutations in the gap junction beta 1 gene (*GJB1*) coding for connexin 32 protein. In addition to typical peripheral neuropathy, central nervous system (CNS) involvement in patients with CMTX1 has been reported as a special feature, but female patients are rarely affected.

**Case presentation:** We describe a 29-year-old female who had a history of two cesarean deliveries. After each delivery, she presented transient and recurrent slurred speech and limb weakness. Magnetic resonance imaging (MRI) showed diffuse abnormal signals in the corpus callosum, posterior limbs of bilateral internal capsule, and centrum semiovale. Electromyogram showed sensorimotor peripheral neuropathy with the characteristics of intermediate CMT. The C.622G>A mutation (p.Glu208Lys) in the *GJB1* gene was detected by PCR-sequencing.

**Conclusion:** The diagnosis of CMTX1 should be considered, even in female patients, when the disease presents with recurrent stroke-like symptoms and abnormal white matter signals on MRI. The puerperium after delivery may be one of the precipitating factors.

## Introduction

Charcot-Marie-Tooth (CMT) is the most common inherited peripheral neuropathy and represents a group of heterogeneous chronic motor-sensory neuropathies. The typical clinical presentation of CMT is characterized by slowly progressive weakness/atrophy in the distal extremities and stocking glove sensory loss. Most patients present pes cavus deformities as an indicated feature. The inheritance patterns of CMT are known to be autosomal dominant inheritance, autosomal recessive inheritance, and X-linked inheritance. Distinct genetic variations may lead to diverse pathophysiologic mechanisms; therefore, CMT is classified into dozens of subtypes according to genetic loci (1).

X-linked Charcot-Marie-Tooth type 1 (CMTX1) is the second most frequent type of CMT (2), which is caused by mutations in the gap junction beta 1 gene (*GJB1*) on chromosome Xq13.1 encoding the connexin 32 (Cx32) protein. Cx32, as a member of gap junction channels (GJCs) protein, forms transmembrane channels to transfer small molecules and ions and is crucial in the maintenance of cellular homeostasis. Hemizygous males are usually affected, while heterozygous CMTX1 females have been reported more frequently than “rarely” and sometimes may be severely affected.

Here, we report a female case who had recurrent stroke-like attacks as the main clinical manifestations accompanied by abnormal white matter signals on MRI and missense mutation in *GJB1*.

## Case Presentation

A female patient, 29 years old, was admitted to our hospital with the chief complaint of “recurrent slurred speech and right limb weakness for 1 day.” One day prior to admission, at 5 a.m. after getting up, she noted an abrupt onset of unclear speech and unstable walking due to weakness of her right limb, which was completely relieved after 3 h. Afterwards, similar symptoms recurred three times, and each episode lasted for ~1–3 h. At 1 p.m. on the day of admission, the symptoms developed into speech difficulties and complete paralysis of the right limbs, which persisted without improvement. In our emergency department, her brain computed tomography (CT) examination revealed no abnormalities. She was clinically diagnosed with ischemic stroke and taken to our neurology department for further treatment.

Past History: 21 days before onset, the patient underwent cesarean section under lumbar anesthesia to give birth to her second child, a healthy baby girl, and the production process was smooth. Remarkably, 7 years ago, she had similar symptoms after she gave birth to a first-born girl by cesarean section. About half a month after delivery, transient slurred speech and right limb weakness recurred approximately three times and then improved quickly and completely. At that time, her brain CT was normal, and no brain MRI was performed. Family history: Her mother died more than 10 years ago with undetermined etiology. Her father was in good health and rejected further genetic investigations. She had no brothers or sisters.

Neurological examination revealed dysarthria, tongue deviation to the right, right upper, and lower limb weakness with muscle strength MRC grade 1, absent deep tendon reflexes in each limb, positive bilateral Babinski sign, bilateral pes cavus, no obvious muscle atrophy, and sensory impairment. Her routine hematological, biochemical, immunological, and thyroid function tests were normal. Moreover, laboratory results for lactate, vitamin B12, and very long chain fatty acids were also within normal ranges.

**Figure 1:**
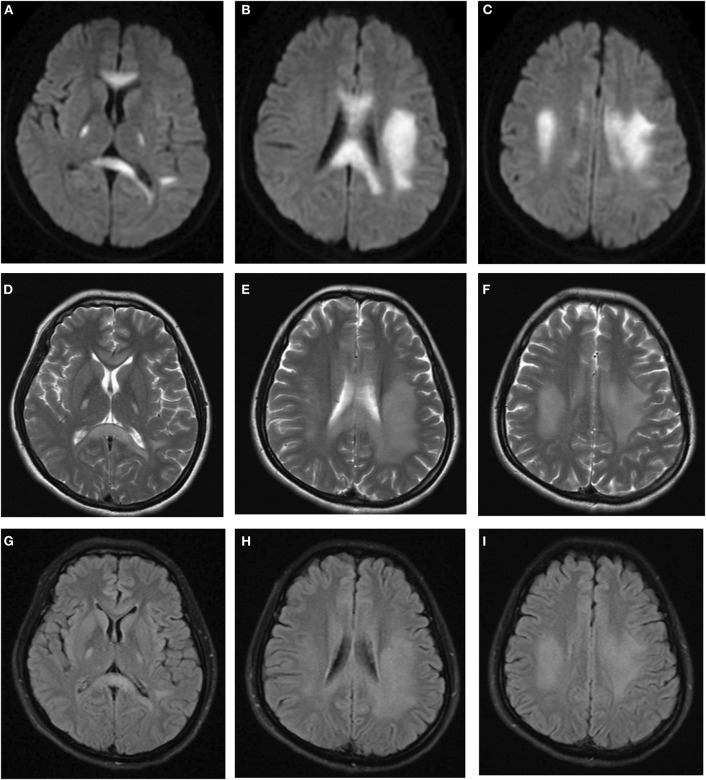
Brain MRI indicated diffuse abnormal signals in corpus callosum, posterior limbs of bilateral internal capsule, and centrum semiovale. **(A–C)** Diffusion weighted imaging. **(D–F)** T2-weighted imaging. **(G–I)** FLAIR imaging.

After admission, she underwent brain magnetic resonance imaging (MRI), showing the diffuse abnormal signal in the corpus callosum, posterior limbs of bilateral internal capsule and centrum semiovale (Figure 1). Further imaging findings of magnetic resonance angiography (MRA) and magnetic resonance venography (MRV) did not support the diagnosis of cerebrovascular disease. Her physical examination (areflexia) indicated that she had peripheral nerve involvement, and therefore electromyogram was further performed. The results showed that the patient had sensorimotor peripheral neuropathy. Moreover, the compound muscle action potential (CMAP) amplitudes of the ulnar, median, tibial and peroneal nerves were normal or slightly attenuated. In contrast, the motor conduction velocities of these nerves were reduced between 32.8 (ulnar nerve) and 18% (tibial nerve) (Table 1). In short, these parameters are characteristics of intermediate CMT (3).

**Table 1:** Nerve conduction study.

**Nerve**	**Segment**	**Latency (ms)**	**Amplitude (sensory in μV, motor in mV)**	**Velocity (m/s)**
**MOTOR**				
Right ulnar	Elbow-Wrist	7.46	6.8	39.5 (↓32.8%)
Right median	Elbow-Wrist	8.65	5.6	40.5 (↓29.9%)
Right tibial	Popliteal fossa-Ankle	13.2	2.4	39.9 (↓18%)
Right common peroneal	Fibular head-Ankle	10.9	1.9	39.5 (↓21%)
**SENSORY**				
Right ulnar	Finger V-Wrist	2.81	3.2	40.9 (↓29.5%)
Right median	Finger III-Wrist	3.48	2.3	41.7 (↓32.7%)
Right sural nerve	Ankle-Middle shin	2.61	2.8	46.0

**Figure 2:**
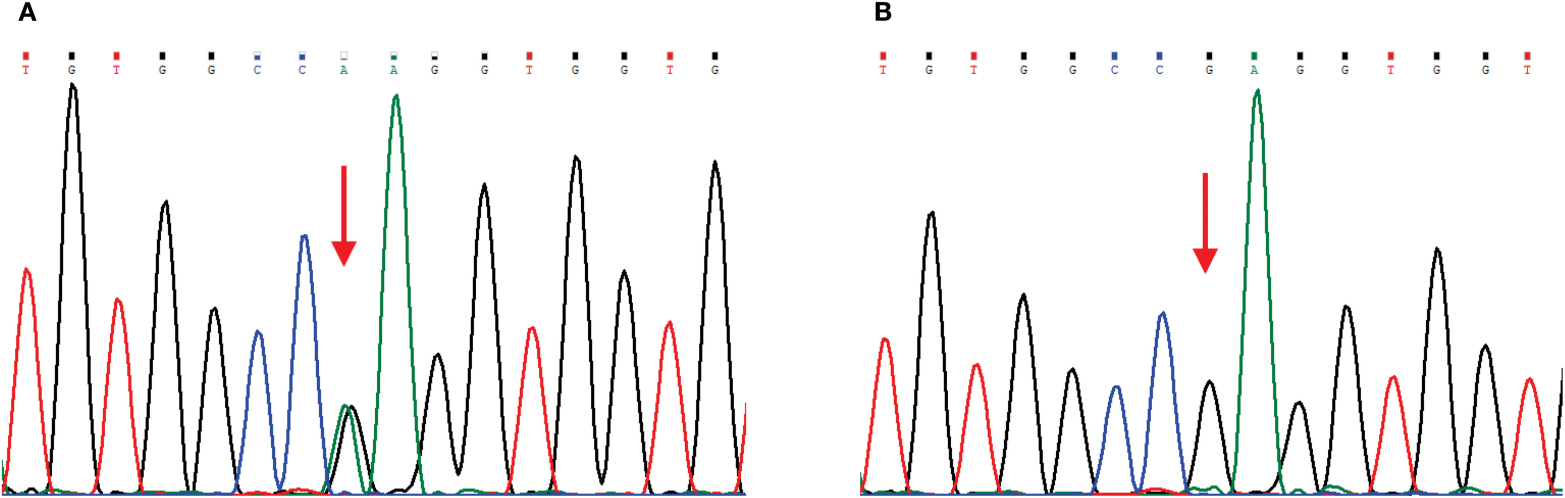
Chromatograms of a *GJB1* mutation. Red arrows indicate the mutation site. **(A)** Sequencing chromatogram of the proband showing heterozygous missense mutation c.622G>A, (p.Glu208Lys) in *GJB1*. **(B)** Sequencing chromatogram of the proband's husband is wild-type.

The main clinical symptom of this patient was central nervous system (CNS) dysfunction, but she had pes cavus deformities, and electrophysiological examination showed peripheral nerve involvement. Although she had no definite family history, we still suspected the possibility of CMTX1. Therefore, we sequenced the *GJB1* gene and confirmed a c.622G>A (p.Glu208Lys) mutation (Figure 2). In the hospital, the patient was conservatively treated without steroids or other surgical procedures. When she was discharged from the hospital (12 days after admission), the muscle strength of her right limb recovered to grade 4. At 1 month of follow-up, the patient's muscle strength returned to normal. Since then, we followed the patient for 3 years, and there was no later onset of stroke-like symptoms.

## Discussion

The main clinical symptom of this reported female case was recurrent CNS neurological deficits, and MRI showed abnormal white matter signals. Finally, CMTX1 was diagnosed by *GJB1* gene analysis.

CMT patients typically present with peripheral neuropathies; in addition, CNS involvement in some CMTX1 patients has been reported as a special feature. The CNS symptoms tend to be acute or subacute onset, last for minutes to days. The clinical manifestations of CNS include dysarthria, hemiparesis, tetraparesis, dysphasia, and numbness. Additionally, ataxia, confusion, and coma have also been reported (4, 5). Meanwhile, in most cases, MRI showed abnormal confluent T2/FLAIR/DWI hyperintensities frequently in the post white matter and corpus callosum (6). These clinical features sometimes mimicked stroke attacks as well as posterior reversible encephalopathy syndrome (PRES) just like this postpartum female patient we reported. In our case, the absent deep tendon reflexes and bilateral pes cavus indicated peripheral nerve involvement. Further electromyogram showed the characteristics of CMTX1 and led to the right diagnosis. Thus, it is important to conduct thorough neurological examination and electrophysiological tests for CMT features in young patients with unexplained stroke-like events and abnormal white matter signals on MRI. In addition, several stress-induced metabolic diseases might cause CNS involvement, such as mitochondrial disease, B12 deficiency, adrenoleukodystrophy, and vanish white matter. However, the results of the images, the normal values of vitamin B12, lactate and very long chain fatty acids, and a 3-year clinical follow-up allowed us to rule out other causes of CNS involvement.

The exact mechanism of CNS dysfunction in CMTX1 is not yet clear. Cx32 is expressed not only in myelinated Schwann cells in the peripheral nerve system (PNS) but also in oligodendrocytes and astrocytes of the CNS. Most studies suggest that these *GJB1* mutations could cause loss of function of Cx32. The abnormal Cx32 may lead to inhibition of transmembrane channel assembly/pore formation, a decrease in the luminal diameter of the channel, or be more sensitive to acidification-induced closure (7–9). For our patient, the c.622G>A mutation was detected using PCR-sequencing, which was previously reported as a mutation associated with CMTX1 (10). To the best of our knowledge, this is the first report about the association of this mutation with CNS involvement and recurrent stroke-like syndrome. Mechanistically, the oligomerization ability of the Glu208Lys mutant was shown to be normal, but its transport capacity to the plasma membrane was decreased. The mutants were retained in the endoplasmic reticulum, and the formation of gap junction was affected (11). The disrupted gap junction communication between astrocytes and oligodendrocytes may result in abnormal fluid exchange and ultimately lead to intramyelinic edema, which may explain the restricted diffusion in the patient's MRI.

CMTX1 is an X-linked dominant hereditary mode in which male patients generally show a uniform phenotype, but female carriers exhibit various phenotypes ranging from asymptomatic, mild to severe symptoms (12). Moreover, female cases with CNS symptoms in CMTX1 are particularly rare. There were occasional instances of CMTX1 females showing subclinical involvement, such as extensor plantar response, abnormal auditory brainstem potential, and brain MRI (13), but none developed acute transient CNS dysfunction (6). This phenomenon could be explained as a result of X-inactivation. Additionally, the patterns of X inactivation vary between different types of cells or tissues (14). Here, we reported an adult-onset female case with CMTX1, who had two episodes occurring at the age of 21 and 29. In this case, the clinical symptoms are mainly concentrated in stroke-like attacks, rather than peripheral polyneuropathy, although we can detect areflexia and pes cavus, suggesting that the pattern of X inactivation in oligodendrocytes of the CNS may be different from that in Schwann cells of the PNS even in the same patient.

It is noteworthy that each of her episodes occurred just after cesarean section, and no such cases have been reported in the past. Previous papers showed that some conditions might precipitate CNS symptoms in patients with CMTX1, such as hyperventilation, febrile illness, intense exercise, concussion or altitude change (15–17). However, how does the puerperium after cesarean delivery trigger CNS symptoms in the female patient with CMTX1? We speculated that hormonal changes that physiologically occur in puerperium (abrupt drop in estrogen and progesterone levels) would hamper the proliferation and maturation of oligodendrocyte progenitor cells to mature oligodendrocytes (18–20). Thus, the female patient with CMTX1 who had fragile oligodendrocyte gap junction coupling due to Cx32 mutation may be more vulnerable in puerperium, leading to recurrent stroke-like symptoms. Further functional studies are required to verify the hypothesis and explore the underlying biological mechanism.

In conclusion, this case highlights that the diagnosis of CMTX1 should be considered when the disease presents with transient or recurrent episodes of CNS neurological deficits and abnormal white matter signals on MRI, even for female patients. The puerperium after cesarean delivery may be a possible precipitating factor for CMTX1.

## Data Availability Statement

The datasets supporting the conclusions of this article are included within the article.

## Ethics Statement

All procedures were approved by the ethics committee of the First Affiliated Hospital of China Medical University. Our patient provided written informed consent.

## Author Contributions

XL and YR had full access to all data in the study and take responsibility of the integrity of the data and the accuracy of the data analysis, study concept and design, drafting of the manuscript, and study supervision. XL and QL collected and analyzed patient information and wrote the manuscript. CC performed the genetic analyses. All the authors read and approved the manuscript.

### Conflict of Interest

The authors declare that the research was conducted in the absence of any commercial or financial relationships that could be construed as a potential conflict of interest.
